# Voronoi Diagram and Crowdsourcing-Based Radio Map Interpolation for GRNN Fingerprinting Localization Using WLAN

**DOI:** 10.3390/s18103579

**Published:** 2018-10-22

**Authors:** Yongliang Sun, Yu He, Weixiao Meng, Xinggan Zhang

**Affiliations:** 1 School of Computer Science and Technology, Nanjing Tech University, Nanjing 211816, China; 2 College of Electronic and Optical Engineering & College of Microelectronics, Nanjing University of Posts and Telecommunications, Nanjing 210023, China; hy1995cool@163.com; 3 School of Electronics and Information Engineering, Harbin Institute of Technology, Harbin 150001, China; wxmeng@hit.edu.cn; 4 School of Electronic Science and Engineering, Nanjing University, Nanjing 210023, China; zhxg@nju.edu.cn

**Keywords:** fingerprinting localization, crowdsourcing, interpolation, Voronoi diagram, general regression neural network

## Abstract

In the last decade, fingerprinting localization using wireless local area network (WLAN) has been paid lots of attention. However, this method needs to establish a database called radio map in the off-line stage, which is a labor-intensive and time-consuming process. To save the radio map establishment cost and improve localization performance, in this paper, we first propose a Voronoi diagram and crowdsourcing-based radio map interpolation method. The interpolation method optimizes propagation model parameters for each Voronoi cell using the received signal strength (RSS) and location coordinates of crowdsourcing points and estimates the RSS samples of interpolation points with the optimized propagation model parameters to establish a new radio map. Then a general regression neural network (GRNN) is employed to fuse the new and original radio maps established through interpolation and manual operation, respectively, and also used as a fingerprinting localization algorithm to compute localization coordinates. The experimental results demonstrate that our proposed GRNN fingerprinting localization system with the fused radio map is able to considerably improve the localization performance.

## 1. Introduction

With the rapid development of information technology, especially the emergence of electronic maps, location-based service (LBS) has been one of current research hot-spots and has been applied to various aspects of people’s daily life, bringing great convenience to people [1]. As a core technology of LBS, localization technology has attracted more and more concerns. Meanwhile, the increasing demands for LBS have prompted the vigorous development of localization technology [2,3]. In outdoor environments, global navigation satellite system (GNSS) plays a vital role in high precision localization, navigation, timing and so on [4]. To offer satisfactory LBS in indoor environments, such as airports, museums, shopping malls, and underground car parks, many technologies have been applied to indoor localization like infrared, ultrasound, ZigBee, ultra-wide band (UWB), radio frequency identification (RFID), inertial sensors, and wireless local area network (WLAN) [2,5,6,7]. Considering the establishment cost and localization performance, WLAN fingerprinting localization has been favored among the existing indoor localization techniques.

The basic WLAN fingerprinting localization method consists of two stages: the off-line and on-line stages [8]. In the off-line stage, several specific locations called reference points (PRs) in the target region are selected. The location coordinates are recorded and received signal strength (RSS) samples are measured at each RP by professional technicians, and then these data are compiled into a database, namely radio map. In the on-line stage, when an RSS sample is measured by a user’s mobile device, localization results can be computed by a fingerprinting localization algorithm. The fingerprinting localization algorithms can be generally categorized into two classes: similarity measurement-based and machine learning-based algorithms. The similarity measurement-based algorithms, for example, K nearest neighbors (KNN) and weighted KNN (WKNN) [9], first measure the similarities between the RSS samples of RPs in the radio map and measured RSS sample in the on-line stage and then estimate the location coordinates of the user with the location coordinates of RPs. Machine learning-based fingerprinting algorithms, such as fuzzy logic, multi-layer perceptron (MLP), and support vector regression (SVR) [8,9,10], calculate localization results with a nonlinear function, which is trained in the off-line stage with the radio map. In this paper, we utilize a general regression neural network (GRNN) to fuse different radio maps and also use it as the fingerprinting algorithm because of its superior performance for function approximation.

However, one disadvantage of the fingerprinting localization method is that the process of establishing the radio map is labor-intensive and time-consuming [11]. Furthermore, the changes of building structure and furniture as well as human movement may cause RSS variations. Consequently, the established radio map needs to be updated at intervals, but the burden of updating the radio map continuously with the professional technicians’ manual operation is difficult to bear. A concept called crowdsourcing has been proposed and then applied to radio map establishment, in which common users, namely crowdsourcing participants, take mobile devices that are able to sense and compute, as well as collectively share, the measured location-labeled RSS data in a participatory manner [12]. Therefore, crowdsourcing has been considered as a solution to the radio map establishment and update of WLAN fingerprinting localization for saving the labor and time cost. The establishment of radio maps through crowdsourcing usually needs a large number of RSS data collected by crowdsourcing participants to calculate an accurate localization result [13].

In this paper, we also apply crowdsourcing to radio map establishment. In the target region, we select a few locations as crowdsourcing points (CPs) and attach two-dimensional (2-D) code stickers at these CPs on the ground. These 2-D code stickers are near the toilet, elevator, and exits, where it is convenient for the crowdsourcing participants to obtain the location coordinates of CPs through scanning these 2-D code stickers. Thus, the crowdsourcing participants are able to collect the RSS data and location coordinates of these CPs and then upload these location-labeled RSS data to a localization server for radio map establishment through interpolation. Using a Voronoi diagram, the target region can be partitioned into Voronoi cells according to the locations of CPs and the propagation model parameters are optimized with crowdsourcing data in each cell so that more accurate RSS data can be estimated. In this way, the proposed Voronoi diagram and crowdsourcing-based radio map interpolation method reduces the labor and time cost for radio map establishment greatly compared with the traditional radio map establishment method using manual operation. To the best of our knowledge, little literature has combined Voronoi diagram and crowdsourcing for radio map establishment.

In this paper, we propose a Voronoi diagram and crowdsourcing-based radio map interpolation method for GRNN fingerprinting localization using WLAN to offer accurate localization results for users in indoor environments. Although the disadvantage of this work is that it is based on a basic fingerprinting localization system with an original radio map, it not only needs no extensive time and labor cost to establish a new radio map, but also achieves a comparable localization performance. The main contributions of this paper can be summarized as follows:

1. We propose an interpolation method for radio map establishment based on a Voronoi diagram and crowdsourcing. The method first partitions the target region into Voronoi cells according to the locations of CPs using a Voronoi diagram. The propagation model parameters in each Voronoi cell are optimized with the RSS data and location coordinates of CPs. Then the RSS data of selected interpolation points (IPs) in each Voronoi cell are estimated with the optimized propagation model parameters and are calibrated according to the RSS data of CPs. So a new radio map can be established through the proposed interpolation method.
2. We propose a GRNN-based fingerprinting localization algorithm, which fuses the two radio maps, consisting of the RSS data and location coordinates of the RPs and IPs, respectively. Then a nonlinear function between the RSS data and location coordinates is approximated by the GRNN using the fused radio map. In the on-line stage, the nonlinear function is used to compute the localization coordinates.
3. We verify the proposed localization system with the RSS data and location coordinates collected from a real indoor environment. The experimental results show that our proposed Voronoi diagram and crowdsourcing-based radio map interpolation method for GRNN fingerprinting localization system is effective in saving radio map establishment cost and improving localization performance.

The remainder of this paper is organized as follows: Section 2 introduces related works including radio map establishment methods and fingerprinting localization algorithms. In Section 3, we first describe the overview of the proposed WLAN fingerprinting localization system, then we discuss the key components of the proposed fingerprinting localization system, which are Voronoi diagram-based region partition, propagation model optimization for interpolation, RSS calibration and GRNN fingerprinting algorithm. Section 4 presents the experimental setup, results, and analyses. Finally, the conclusions and future work are given in Section 5.

## 2. Related Works

### 2.1. Radio Map Establishment Methods

One of the key components of WLAN fingerprinting localization is to establish the radio map, so people have paid great attention on it. Currently, most of the research on radio map establishment is based on crowdsourcing. P. Bolliger [14] first proposed that location-labeled RSS data could be collected using an electronic map. Then crowdsourcing-based radio map establishment methods using electronic maps were also presented in Ref. [15,16]. C. Wu et al. [17] measured RSS data and trajectories of crowdsourcing participants and matched these RSS data with trajectories using multidimensional scaling for radio map establishment. Additionally, P. Mirowski et al. [18] proposed that crowdsourcing participants could obtain location coordinates for RSS data through scanning the 2-D code labels deployed in the experimental region. In Ref. [19], location information could be measured near building entrances and windows using global positioning system (GPS) and then used to label the collected RSS data. S. Jung and D. Han [20] proposed a radio map establishment and maintenance system using cowdsourced RSS data collected from numerous mobile devices. The system exploited an unsupervised learning algorithm to establish an initial radio map. Then the radio map was adapted according to the RSS variations through crowdsourcing. L. Ma et al. [21] obtained location information of RSS data using crowdsourcing pedestrian dead reckoning (PDR) trajectories for radio map establishment. They transformed the locations in PDR trajectories from relative ones to absolute ones through trajectory matching using Hough transform and Harris corner detection. N. Yu et al. [22] exploited crowdsourcing RSS data to identify the indoor doors that were regarded as reference locations. Then the representative fingerprints were extracted and linked to their corresponding physical locations with the reference locations and RSS similarities. In this way, a radio map could be established.

In addition to the above-mentioned crowdsourcing method for radio map establishment, other radio map establishment methods have also been proposed. J. Yin et al. [23] reconstructed a radio map by using real-time RSS data at RPs. They took real-time RSS values at each time point into account and utilized the dependency between the estimated locations and RPs. H. Wang et al. [24] proposed a dynamic radio map establishment method for WLAN fingerprinting localization. In the off-line stage, the relationship between RSS data of calibration points and RPs was established by an MLP. In the on-line stage, the real-time RSS values at RPs were predicted based on the RSS data collected at calibration points. C. Zhou and Y. Gu presented a joint method of indoor localization and radio map establishment in Ref. [25]. They exploited the inherent spatial correlation of RSS measurements to reduce the calibrating RSS data and perform localization without a full radio map. The accumulation of localization information could be jointly used to establish the radio map. In Ref. [26], H. Zhao et al. considered the comprehensive features of RSS data by adopting universal Kriging method and region partitioning. Naive Bayes classifier and WKNN were used to work with the interpolated radio map. M. Lee and D. Han [27] proposed an interpolation method based on a Voronoi diagram. The method refined the propagation model of each cell for RSS estimation considering the signal fading caused by walls. Compared with their method, through using crowdsourcing, we partition the experimental environment according to the locations of CPs. The estimated RSS data can be updated according to the RSS variations in the indoor environment and the Voronoi cells can be easily adapted according to the locations of CPs.

### 2.2. Fingerprinting Localization Algorithms

Fingerprinting localization algorithm is essential to WLAN fingerprinting localization. Besides the fingerprinting algorithms referred to above, like the similarity measurement-based algorithms KNN and WKNN, fingerprinting algorithms based on cosine similarity [28] and Pearson correlation coefficient [29] also measure the similarities between the RSS samples stored in the radio map and those collected in the on-line stage. Then these algorithms compute localization results according to selected RP location coordinates whose RSS samples are similar to those collected in the on-line stage. The drawbacks of the similarity measurement-based algorithms are not only the localization performance can be influenced by the RP density and distribution, but also the algorithms suffer high computation complexity if the radio map has a large number of RPs. Regarding the machine learning-based fingerprinting algorithms, machine learning algorithms like radial basis function (RBF) neural network [30] and adaptive neuro-fuzzy inference system (ANFIS) [31] could also be used as fingerprinting algorithms. In the off-line stage, these fingerprinting algorithms are trained with the radio map to approximate to a nonlinear function between RSS data and location coordinates. In the on-line stage, when a mobile device measures an RSS sample, the RSS sample is inputted into the trained nonlinear function and then location coordinates of the mobile device can be estimated with the nonlinear function.

In recent years, many advanced fingerprinting localization algorithms have been proposed. J. Talvitie et al. [32] proposed an RSS-based indoor localization method using RSS images and compressed spectral analysis. The method was able to considerably reduce the size of the radio map only by storing the most significant coefficients from discrete cosine transformed images. An efficient and accurate localization method called Tilejunction was proposed in Ref. [33]. The method mapped the user RSS data of each access point (AP) to a convex hull termed signal “tile”. Then it used a linear programming method to locate the user at the junction of the tiles. X. Wang et al. [34] presented a deep learning-based indoor fingerprinting localization system using channel state information. In the off-line stage, deep learning was utilized to train all the weights of a deep network. In the on-line stage, a probabilistic method was used to compute the location. K. Chen et al. [35] presented a fingerprinting method called Slide that could achieve automatic mapping from the RSS data to the locations on a line. The method also acquired the locations after each WLAN channel scanning to mitigate the misalignment problem. In Ref. [36], an indoor localization system using WLAN named HybLoc was proposed. The system utilized Gaussian mixture model-based soft clustering and random decision forest for localization.

In short, fingerprinting localization can be formulated as a regression problem that is to predict location coordinates with RSS data using an approximated nonlinear function. Because GRNN has a superior regression performance and appropriate noise attenuation performance, which is usually used for function approximation [37], we apply a GRNN as the fingerprinting algorithm in this paper to compute localization coordinates.

## 3. Proposed Localization System

### 3.1. System Overview

**Figure 1:**
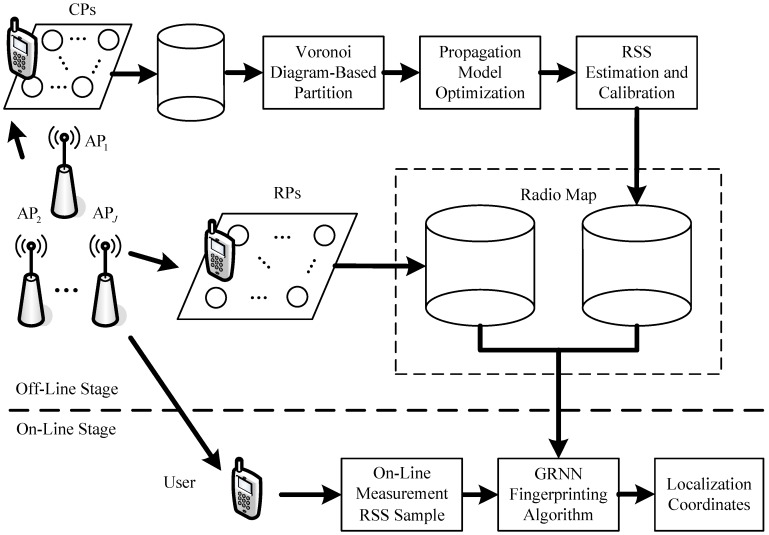
Diagram of the proposed fingerprinting localization system.

As the basic fingerprinting localization system, our proposed localization system can also be generally divided into two stages: the off-line stage and on-line stage, which is shown in Figure 1. We apply crowdsourcing to the fingerprinting localization system so that the established radio map through interpolation method can be updated continuously according to the RSS variations in the indoor environment.

In the off-line stage, we also establish a radio map with the RSS data and location coordinates of RPs in the indoor environment, which we consider the original radio map. We select a few extra locations as the CPs and attach 2-D code stickers with location information printed on them, so that the crowdsourcing participants can scan and obtain the location coordinates to label the RSS data measured at these CPs. We exploit the Voronoi diagram to partition the indoor environment into small Voronoi cells. In each Voronoi cell, there is only one CP and the RSS data and location coordinates of the CP are used for optimizing propagation model parameters. We compute the mean value of each propagation model parameter for interpolation. After the locations of IPs are determined around the CP and RPs in the Voronoi cell, the RSS values of different APs are estimated for the IPs with the optimized propagation model parameters and then calibrated with the RSS data of the CP. When the computation and calibration of RSS data at all the IPs are finished, a new radio map is established through interpolation. Then, we utilize a GRNN to fuse the two radio maps and approximate to a nonlinear function $F:{\mathbb{R}}^{J}\to {\mathbb{R}}^{2}$ between the RSS data and location coordinates. We take the off-line RSS data $RSS_{i}=\left\{RSS_{i,1},RSS_{i,2},\cdots ,RSS_{i,J}\right\},i\in \left\{1,2,\cdots ,I\right\}$ as the inputs of the GRNN and the corresponding location coordinates $L_{i}=\left(x_{i},y_{i}\right),i\in \left\{1,2,\cdots ,I\right\}$ as its outputs, where I and J denote the numbers of training samples and deployed APs, respectively.

In the on-line stage, when the user takes a mobile device and measures an on-line RSS sample $rss=\left\{rss_{1},rss_{2},\cdots ,rss_{J}\right\}$, the on-line RSS sample is inputted into the GRNN. Then localization coordinates $\;\hat{l}\;=\left(\hat{x},\hat{y}\right)$ can be computed by $\;\hat{l}\;=F\left(rss\right)$. This stage of the proposed localization system is the same as the basic fingerprinting localization system.

### 3.2. Voronoi Diagram-Based Region Partition

A Voronoi diagram is also known as a Thiessen polygon or Dirichlet tessellation, which offers an effective solution for partitioning a plane into a few regions [38]. It has been used in various fields like local coverage optimization of wireless sensor network [39], interferometric synthetic aperture radar (InSAR) fine registration [40], and optimal allocation of dynamic reactive power sources [41]. The general idea of the Voronoi diagram is to partition the plane into regions according to the locations of a set of sites. Each region is called a Voronoi cell and usually there is one site in each Voronoi cell. Any other points in the cell is closer to the corresponding site in the same cell than to other sites. Based on Delaunay triangulation, the perpendicular bisectors for each Delaunay line can be determined, which can intersect at an endpoint called Voronoi vertex. The line segments between Voronoi vertexes are the Voronoi edges and the convex polygon region surrounded by the Voronoi edges is the Voronoi cell.

We take the CPs as the sites and let $\xi =\left\{c^{\left(1\right)},c^{\left(2\right)},\cdots ,c^{\left(K\right)}\right\}$ denote the set of CP locations in the experimental environment ${\mathbb{R}}^{2}$, where $c^{\left(k\right)}=\left(x_{\mathrm{CP}}^{\left(k\right)},y_{\mathrm{CP}}^{\left(k\right)}\right),k\in \{1,2,\cdots K\}$, and $r^{\left(l\right)}=\left(x_{\mathrm{IP}}^{\left(l\right)},y_{\mathrm{IP}}^{\left(l\right)}\right)$ denote the location coordinate vector of lth IP. The lth IP in the experimental environment whose distance to kth CP $c^{\left(k\right)}$ are less than or equal to the distances to other CPs $c^{\left(i\right)},i\neq k$, is in the kth Voronoi cell $V_{k}$, then the kth Voronoi cell $V_{k}$ can be denoted as follows:

(1) $$V_{k}=\left\{r^{\left(l\right)}\in {\mathbb{R}}^{2}|d^{\left(k,l\right)}\leq d^{\left(i,l\right)},\forall c^{\left(i\right)}\in \xi ,i\neq k\right\}\;$$

where, $d^{\left(k,l\right)}$ and $d^{\left(i,l\right)}$ denote the Euclidean distances between kth CP and lth IP and between ith CP and lth IP, respectively, which can be calculated by:

(2) $$d^{\left(k,l\right)}=\sqrt{{\left(x_{\mathrm{CP}}^{\left(k\right)}-x_{\mathrm{IP}}^{\left(l\right)}\right)}^{2}+{\left(y_{\mathrm{CP}}^{\left(k\right)}-y_{\mathrm{IP}}^{\left(l\right)}\right)}^{2}}\;$$

(3) $$d^{\left(i,l\right)}=\sqrt{{\left(x_{\mathrm{CP}}^{\left(i\right)}-x_{\mathrm{IP}}^{\left(l\right)}\right)}^{2}+{\left(y_{\mathrm{CP}}^{\left(i\right)}-y_{\mathrm{IP}}^{\left(l\right)}\right)}^{2}}\;$$

### 3.3. Propagation Model Optimization for Interpolation

As we know, because wireless signal attenuates with the increasing propagation distance, RSS data measured in indoor environments are spatially related. When we partition the indoor experimental environment into several Voronoi cells according to the locations of CPs, the RSS data collected in the same Voronoi cell usually have the similar RSS characteristics. This is because the wireless links between the APs and locations in the same Voronoi cell could be influenced by the same factors, such as the building structure, furniture, and presence of humans.

Thus, we optimize propagation model parameters with the known location-labeled RSS data of the CP and calculate the RSS data with the optimized propagation model parameters for the IPs in the same Voronoi cell. In this paper, we adopt a common propagation model [42] to estimate the RSS data for the IPs. The propagation model is denoted as follows:

(4) $$P_{\mathrm{Total}}=P_{\mathrm{AP}}-P_{\mathrm{Dev}}=20\log_{10}f+N\log_{10}d-X\;$$

where, $P_{\mathrm{Total}}$ denotes the total path loss between an AP and mobile device, $P_{\mathrm{AP}}$ denotes the power transmitted by the AP that can be derived from AP configurations, $P_{\mathrm{Dev}}$ denotes the received power that can be derived from the RSS data measured by the mobile device, f denotes the frequency, d denotes the distance between the AP and mobile device, and both N and X denote the propagation model parameters that are usually set equal to 30 and 28 at a frequency of 2.4 GHz in office environments, respectively. These two parameters, N and X, are the propagation model parameters we need to optimize with the crowdsourcing data in each Voronoi cell.

We let $P_{\mathrm{AP}}^{\left(j\right)}$ and $P_{\mathrm{Dev}}^{\left(j,k\right)}$ denote the transmitted power of jth AP and received power at kth CP by the device from jth AP, respectively, $d^{\left(j,k\right)}$ denote the distance between jth AP and kth CP, and $N^{\left(j,k\right)}$ and $X^{\left(j,k\right)}$ denote the propagation model parameters between jth AP and kth CP. Then the total path loss between jth AP and kth CP can be computed by $P_{\mathrm{Total}}^{\left(j,k\right)}=P_{\mathrm{AP}}^{\left(j\right)}-P_{\mathrm{Dev}}^{\left(j,k\right)}$ and we can have (5) as follows:

(5) $$P_{\mathrm{Total}}^{\left(j,k\right)}=20\log_{10}f+N^{\left(j,k\right)}\log_{10}d^{\left(j,k\right)}-X^{\left(j,k\right)}\;$$

The distance $d^{\left(j,k\right)}$ between jth AP and kth CP can be estimated with the propagation model denoted by:

(6) $$d^{\left(j,k\right)}=10^{\frac{P_{\mathrm{Total}}^{\left(j,k\right)}-20\log_{10}f+X^{\left(j,k\right)}}{N^{\left(j,k\right)}}}\;$$

Because the location coordinates of the APs and CPs are known, the real distance between each AP and CP can be computed. We let $a^{\left(j\right)}=\left(x_{\mathrm{AP}}^{\left(j\right)},y_{\mathrm{AP}}^{\left(j\right)}\right)$ and $c^{\left(k\right)}=\left(x_{\mathrm{CP}}^{\left(k\right)},y_{\mathrm{CP}}^{\left(k\right)}\right)$ denote the known location coordinates of jth AP and kth CP, respectively, so the real distance $d_{\mathrm{Real}}^{\left(j,k\right)}$ between jth AP and kth CP can be calculated by (2) or (3). Then the absolute value of the distance difference between the estimated and real distances is calculated by:

(7) $$\left|\Delta d^{\left(j,k\right)}\right|=\left|d^{\left(j,k\right)}-d_{\mathrm{Real}}^{\left(j,k\right)}\right|\;$$

The propagation model parameters $N^{\left(j,k\right)}$ and $X^{\left(j,k\right)}$ are optimized to minimize $\left|\Delta d^{\left(j,k\right)}\right|$ calculated by (7). The process of searching the optimized parameters ${\hat{N}}^{\left(j,k\right)}$ and ${\hat{X}}^{\left(j,k\right)}$ can be formulated as an unconstrained nonlinear programming problem denoted by:

(8) $$\left({\hat{N}}^{\left(j,k\right)},{\hat{X}}^{\left(j,k\right)}\right)=\underset{\left(N^{\left(j,k\right)},X^{\left(j,k\right)}\right)}{\arg\min}\left|10^{\frac{P_{\mathrm{Total}}^{\left(j,k\right)}-20\log_{10}f+X^{\left(j,k\right)}}{N^{\left(j,k\right)}}}-d_{\mathrm{Real}}^{\left(j,k\right)}\right|\;$$

In this paper, the initial values of the propagation model parameters $N^{\left(j,k\right)}$ and $X^{\left(j,k\right)}$ are set equal to 30 and 28, respectively. We adopt Nelder-Mead simplex algorithm [43] for the parameter optimization. In each Voronoi cell, multiple RSS samples collected from different APs are used to optimize the two propagation model parameters. So multiple optimized parameters can be obtained. We calculate the mean values of the two parameters and utilize the mean values to predict the RSS data at IPs.

### 3.4. RSS Calibration and GRNN Fingerprinting Localization Algorithm

#### 3.4.1. RSS Calibration

Due to the blockage of walls, doors, furniture and so on, the wireless signals from some APs at some locations may be so weak that they cannot be detected by mobile devices, which causes the corresponding RSS values are unavailable and equal to 0 in the mobile devices. When we estimated RSS values with the optimized propagation model parameters, few estimated RSS values at the IPs are 0. Thus, we need to calibrate the estimated RSS data according to the RSS data of CPs measured in the real environment. For example, if all the RSS values from jth AP at kth CP are 0, which means jth AP cannot be detected by the mobile device at kth CP, then we need to calibrate the jth vector of the RSS samples estimated with the optimized propagation model parameters in kth Voronoi cell. We find such RSS vector using the mean value of the RSS vector ${\bar{\mathrm{rss}}}_{j,k}$ of kth CP from jth AP, which can be computed by:

(9) $$\bar{r}\bar{s}{\bar{s}}_{j,k}=\sum_{i=1}^{I}rss_{i,j,k}\;$$

where, I denotes the number of RSS values from jth AP measured at kth CP. If the RSS mean value ${\bar{\mathrm{rss}}}_{j,k}$ is equal to 0, then we can calibrate the RSS values estimated with the optimized propagation model parameters of jth AP in kth Voronoi cell and set them equal to 0. So these estimated RSS samples can be more similar with the ones measured in the real environment.

#### 3.4.2. GRNN Fingerprinting Localization Algorithm

**Figure 2:**
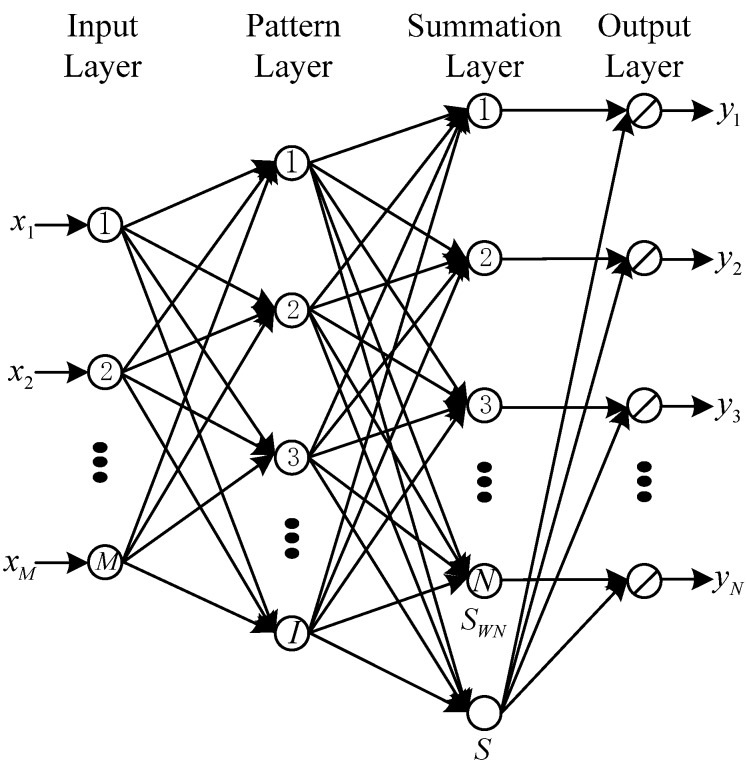
The basic structure of GRNN.

After the RSS calibration, the estimated RSS data, along with the location coordinates of all the IPs, can be compiled into a new radio map. Because GRNN does not need iterative training like the MLP trained by back propagation (BP) algorithm and it has a superior regression performance to MLP or RBF network [44], we use a GRNN to fuse the new radio map established through interpolation and the original radio map that consists of the location-labeled RSS data of RPs and then to approximate a nonlinear function between RSS data and location coordinates. A basic GRNN structure is shown in Figure 2. The GRNN consists of four layers that are the input layer, pattern layer, summation layer, and output layer. In addition, after we obtain the nonlinear function F using the GRNN, the localization coordinates can be calculated in the on-line stage.

As shown in Figure 2, we let $\zeta =\left\{x_{i},y_{i},i=1,2,\cdots ,I\right\}$ denote the training data set, $x=\left\{x_{1},x_{2},\cdots ,x_{M}\right\}$ denote the input vector, and $\;\hat{y}\;=\left\{{\hat{y}}_{1},{\hat{y}}_{2},\cdots ,{\hat{y}}_{N}\right\}$ denote the desired output vector, which can be the fused radio map, an on-line RSS sample measured by a mobile device, and localization coordinates in this paper, respectively. In the input layer, the number of neurons is the same as the number of inputs of the GRNN and all the neurons in the input layer are connected with the neurons in the pattern layer. In the pattern layer, a Gaussian function is used and denoted by:

(10) $$\left\{ \begin{array}{l} g_{i}=\exp\left[-\frac{D_{i}^{2}}{2\sigma^{2}}\right]\\ D_{i}^{2}={\left(x-x_{i}\right)}^{T}\left(x-x_{i}\right) \end{array}\right.\;$$

where, σ denotes a smoothing parameter that can be obtained through cross validation in the off-line stage and $D_{i}^{2}$ denotes the squared distance between x and $x_{i}$.

In the summation layer, there are two kinds of neurons. One kind of neuron calculates the weighted sum $S_{Wn},n=1,2,\cdots ,N$ of the outputs in the pattern layer; the other kind of neuron calculates the sum S of the pattern layer outputs. So we can compute the nth element of the desired output vector as follows:

(11) $$\left\{ \begin{array}{l} {\hat{y}}_{n}=\frac{S_{Wn}}{S}\\ S_{Wn}=\sum_{i=1}^{I}y_{i,n}g_{i}\\ S=\sum_{i=1}^{I}g_{i} \end{array}\right.\;$$

where, ${\hat{y}}_{n}$ and $y_{i,n},n=1,2,\cdots ,N$ denote nth elements of the output vectors $\hat{y}$ and $y_{i}$, respectively. Thus, when a user measures an RSS sample and inputs it into the GRNN in the on-line stage, the localization coordinates can be computed.

## 4. Experimental Setup, Results, and Analyses

### 4.1. Experimental Setup

**Figure 3:**
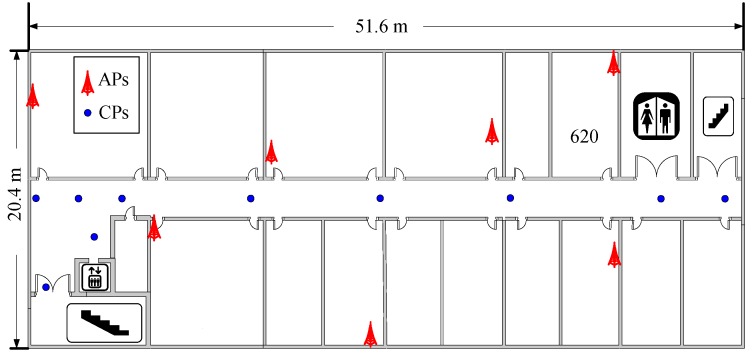
Experimental floor plan.

**Figure 4:**
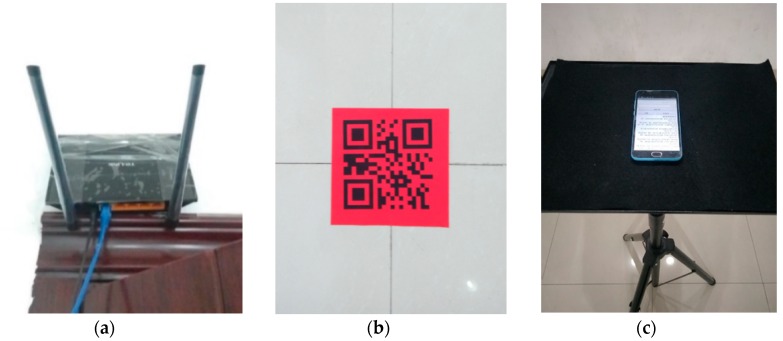
Experimental scenario of WLAN fingerprinting localization: (**a**) TP-LINK TL-WR845N AP; (**b**) 2-D code sticker on the ground; (**c**) Meizu M2 smartphone on a tripod.

In this paper, we collected all the experimental data from a real office environment. The experimental environment was a rectangular region with dimensions of 51.6 m × 20.4 m, as shown in Figure 3. A total of 7 TP-LINK TL-WR845N (TP-LINK Co., Ltd., Shenzhen, China) APs were mounted in the experimental environment at a height of 2.2 m to offer wireless communication service, as shown in Figure 4a. Because the office rooms might not be free to enter for the crowdsourcing participants to collect RSS data, we selected 10 specific locations as CPs only in the corridor, which were marked with the blue points in Figure 3. We labeled the 10 CPs with 2-D code stickers on the ground of the corridor, as shown in Figure 4b, so that the crowdsourcing participants could scan the 2-D codes on the stickers and obtain the location coordinates of the CPs. Because we assumed that the crowdsourcing participants collected the RSS data and location coordinates of the CPs in a voluntary manner, the time for the crowdsourcing participants to collect these data must be less than one minute [45], and only 60 RSS samples were collected at each CP. We selected 92 RPs in the corridor and Room 620, and then collected 120 RSS samples at each RP. To test the proposed fingerprinting localization system, a trajectory consisting of 90 testing points (TPs) with 0.6 m gaps were selected from the end of the corridor near the elevator to Room 620 in order to simulate a person walking along the trajectory in the experimental environment. A total of 5400 RSS samples were collected along the trajectory to test the system. All the RSS data were measured by a Meizu M2 (Meizu Technology Co., Ltd., Zhuhai, China) smartphone that was placed on a tripod at a height of 1.2 m, as shown in Figure 4c. This smartphone was installed with our self-developed Android application software, the sampling rate of which was 1 RSS sample per second.

### 4.2. Experimental Results and Analyses

**Figure 5:**
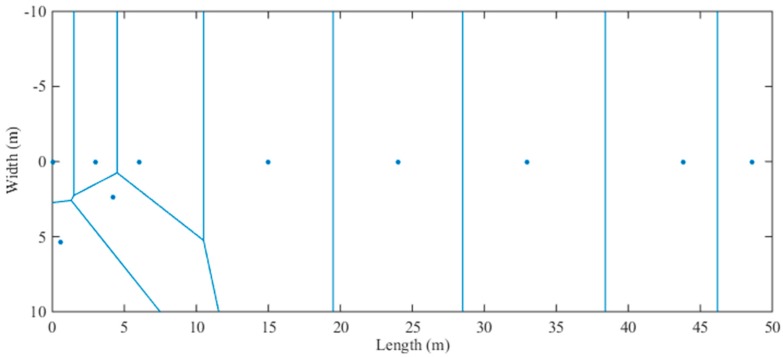
The partitioning result of the experimental environment using the Voronoi diagram.

As mentioned above, we selected 10 CPs in the experimental environment, so the experimental environment is partitioned into 10 regions with a Voronoi diagram according to the locations of the CPs, with each region being a Voronoi cell. The partitioning result is shown in Figure 5, below. Because all of the CPs are in the corridor, most of the Voronoi cells in the straight corridor can be the standard rectangles, and it is easy for us to select the locations of IPs. In each Voronoi cell, we exploit the RSS data and location coordinates of CP to optimize the propagation model parameters between the CP and each AP.

**Figure 6:**
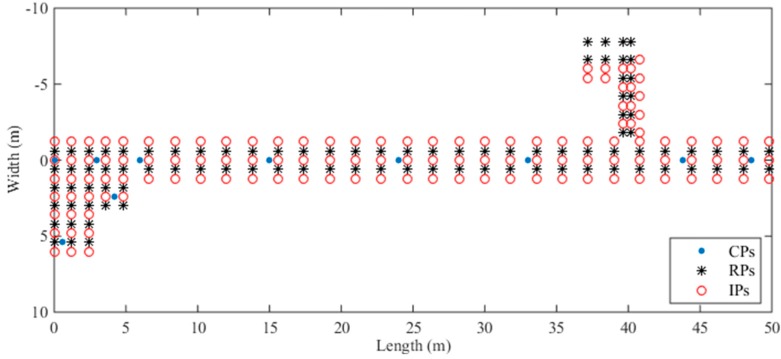
The locations of CPs, RPs, and IPs in the experimental environment.

Then we select 128 specific locations in the experimental environment as IPs and estimate RSS data for these IPs. These IPs are around the RPs and CPs, and the locations of the CPs, RPs, and IP are shown in Figure 6. Specifically, the 128 IPs are distributed in the corridor and Room 620 like the RPs. We estimate the RSS data of different APs for these IPs with the optimized propagation model parameters in each Voronoi cell. Due to the influence of the experimental environment, before we compile these estimated RSS data into a new radio map for localization, we need to calibrate the estimated RSS data for IPs with the RSS data of the CP in the same Voronoi cell that can be collected in the real environment by crowdsourcing participants.

**Figure 7:**
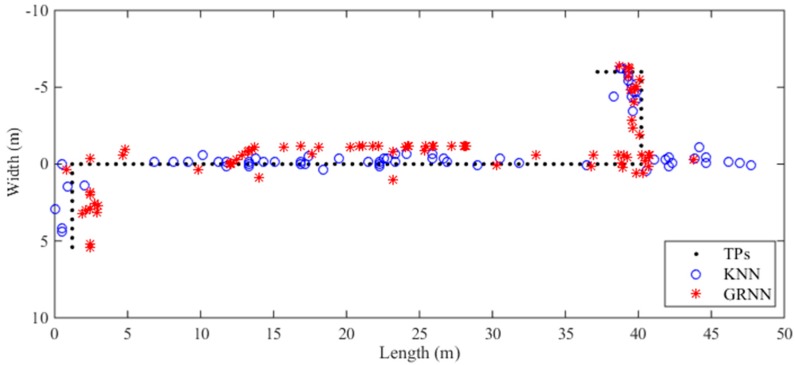
Localization results of one trajectory computed by the KNN and GRNN algorithms.

We first take one trajectory as an example, the localization results with the fused radio map computed by the traditional KNN and proposed GRNN algorithms are shown in Figure 7. In the experiment, we select the 7 nearest RPs for the KNN to compute localization coordinates and utilize 4-fold cross validation for the GRNN to optimize the smoothing parameter σ in the off-line stage. The mean errors of the localization results computed by the KNN and GRNN are 3.57 m and 2.63 m, respectively. As shown in Figure 7, the localization results computed by the GRNN algorithm are more continuous than those computed by the KNN algorithm. Although the localization results of the KNN algorithm are near to the true trajectory in the middle segment of the corridor, the localization results of the KNN algorithm deviate from the real locations of TPs greatly in the region near Room 620. Some of these localization results even reach to the toilet and the stairs.

We also compute the localization results using all the RSS data at TPs and the fused radio map, and then compare the localization performance of the fingerprinting algorithms KNN, WKNN, MLP, and our proposed GRNN. We set the same parameters for the KNN, WKNN, and GRNN as in the simple trajectory experiment. Regarding the MLP we utilize, it is trained by the BP algorithm in the off-line stage. The numbers of neurons in the input layer, hidden layer, and output layer of the MLP are 7, 40, and 2, respectively. The number of iterative epochs is set to be equal to 10,000. Meanwhile, Sigmoid function and linear function are selected as the activation functions for the hidden layer and output layer of the MLP, respectively.

**Figure 8:**
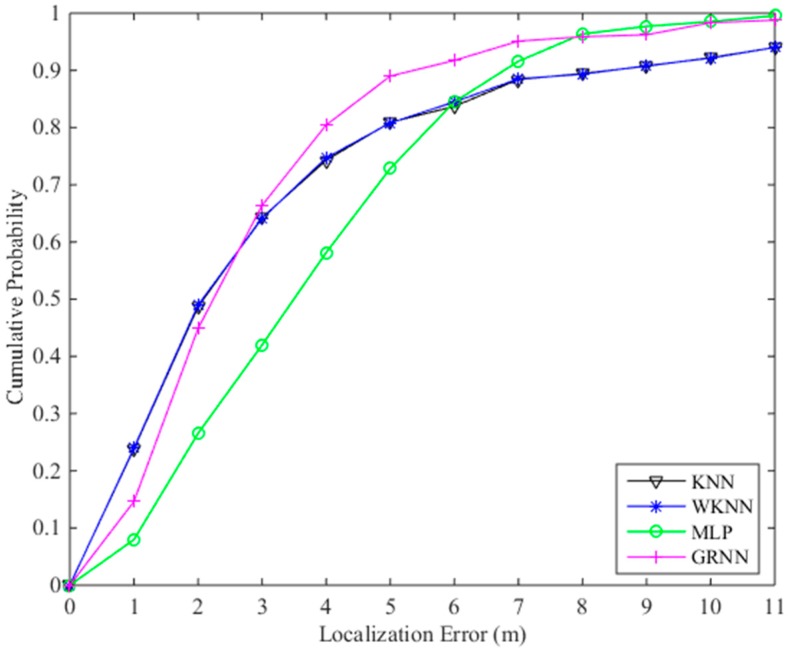
Cumulative probabilities of localization errors computed by different fingerprinting algorithms using the fused radio map.

The localization results are listed in Table 1. The mean errors of localization results computed by the fingerprinting algorithms KNN, WKNN, MLP and GRNN with the fused radio map are 3.29 m, 3.27 m, 3.75 m and 2.78 m, respectively. The cumulative probabilities of the KNN, WKNN, MLP and GRNN within a localization error of 3 m are 64.3%, 64.1%, 42.0% and 66.4%, respectively. The cumulative probabilities of the KNN, WKNN, MLP and GRNN within a localization error of 4 m are 74.3%, 74.7%, 58.2% and 80.4%, respectively. We also test the KNN algorithm using the original radio map that only consists of the RSS data and location coordinates of RPs. The mean error of the KNN using the original radio map is 4.20 m. Obviously, the localization results using the fused radio map are more accurate than those computed using the original radio map. The cumulative probability curves of the KNN, WKNN, MLP and GRNN fingerprinting algorithms are shown in Figure 8. In Figure 8, the cumulative probabilities of the GRNN algorithm within a localization error of 2 m are a little lower than those of the KNN and WKNN algorithms, but larger localization errors are computed by the KNN and WKNN. Generally, the proposed GRNN fingerprinting algorithm outperforms the other three fingerprinting algorithms.

## 5. Conclusions and Future Work

In this paper, we propose a WLAN fingerprinting localization system, which is not only able to save labor and time cost for radio map establishment, but also achieves a comparable localization performance. In the off-line stage, we exploit the Voronoi diagram and crowdsourcing to establish a new radio map through interpolation. We first partition the experimental environment into Voronoi cells using the Voronoi diagram according to the locations of CPs, and then optimize the propagation model parameters with the CP data, so that the estimated RSS data using optimized propagation model parameters after RSS calibration will be more similar to the RSS data measured in the real environment. Because the radio map is established based on crowdsourcing data collected by crowdsourcing participants, it can be easily updated according to the RSS variations in the indoor environment. Owing to the superior performance of GRNN for function approximation, we use a GRNN as a fingerprinting algorithm to fuse the new radio map established through interpolation and the original radio map and to approximate to a nonlinear function between RSS data and location coordinates. Then localization coordinates can be computed using the nonlinear function in the on-line stage. The experimental results show that our proposed fingerprinting localization system can reduce the mean error of localization results to 2.78 m with the crowdsourcing data collected at only a few CPs, which proves the effectiveness of the proposed system. We expect that the proposed system could offer a valuable reference for WLAN fingerprinting localization and promote the theoretical research and practical application of WLAN fingerprinting localization to some degree.

In the future, we intend to continue the work on radio map interpolation to reduce labor and time cost for radio map establishment. We might also focus on the crowdsourcing-based localization performance improvement against device diversity, multi-signal fingerprinting localization, and high-accuracy fingerprinting localization based on deep learning.

## Figures and Tables

**Table 1:** Localization results of different fingerprinting algorithms using the fused radio map.

Algorithm	Mean Error (m)	Cumulative Probability (%)	
		Within 3 m Error	Within 4 m Error
KNN	3.29	64.3	74.3
WKNN	3.27	64.1	74.7
MLP	3.75	42.0	58.2
GRNN	2.78	66.4	80.4
